# A Pragmatic Approach for Brain‐First and Body‐First Classification in Parkinson's Disease: Insights from the Personalized Parkinson Project

**DOI:** 10.1002/mdc3.70913

**Published:** 2026-11-09

**Authors:** Kars I. Veldkamp, Nienke A. Timmermans, Elbrich M. Postma, Rick C. Helmich, Bastiaan R. Bloem, Per Borghammer, Luc J.W. Evers

**Affiliations:** ^1^ Department of Neurology, Center of Expertise for Parkinson and Movement Disorders Donders Institute for Brain, Cognition and Behaviour, Radboud University Medical Center Nijmegen The Netherlands; ^2^ Lundbeck Foundation Parkinson's Disease Research Center (PACE) Aarhus Denmark

**Keywords:** autonomic nervous system, central nervous system, clinical subtypes, Parkinson's disease

## Abstract

**Background:**

The brain‐first versus body‐first framework in Parkinson's disease (PD) proposes distinct origins of α‐synuclein pathology, resulting in discrete progression patterns. Although this pathophysiological distinction implies different clinical trajectories, feasible criteria to classify subjects in large cohorts are currently lacking.

**Objectives:**

The goal was to develop a pragmatic approach for classifying brain‐first and body‐first PD clinical subtypes in a large early‐PD cohort.

**Methods:**

We, therefore, explored a pragmatic approach to separating these subtypes based on motor asymmetry, rapid eye movement sleep behavior disorder, and orthostatic hypotension, applied in a large cohort of persons with early PD (disease duration <5 years).

**Results:**

Individuals classified as probable brain‐first were more often tremor‐dominant and showed a trend toward less constipation, after adjusting for disease duration, age and sex.

**Conclusions:**

This framework enables subtyping in many existing cohorts, potentially advancing our understanding of PD pathophysiology and facilitating targeted therapies. Validation against gold‐standard measures is recommended to refine these criteria further.

Recent evidence suggests the existence of brain‐first and body‐first subtypes of Parkinson's disease (PD), which is distinguished by the origin of α‐synuclein pathology.^1^ This hypothesis suggests that the initial pathology starts in a specific location and eventually spreads to all vulnerable structures in the nervous system. The origin of this pathology varies between individuals, leading to the development of two distinct subtypes at the extremes of the spectrum, with presumably some intermediate subtypes in between. In brain‐first PD, the pathology starts in one hemisphere (olfactory bulb or amygdala), whereas body‐first PD originates in the enteric or peripheral autonomic nervous system, from where the pathology spreads toward the central nervous system via parasympathetic and sympathetic connections. Consequently, brain‐first cases are characterized by more asymmetric neurodegeneration in the brain and later onset of autonomic dysfunction,^2^ whereas body‐first cases exhibit early degeneration of the autonomic nervous system and lower‐to‐middle brainstem structures, followed by more symmetric neurodegeneration in the brain. Distinguishing between these subtypes is important, as they may represent distinct forms of PD with different progression patterns.^3^ However, the absence of harmonized, feasible criteria based on widely accessible and affordable measures currently limits reliable differentiation and hinders further research into their clinical manifestations. Ideally, the distinction between brain‐first and body‐first PD is based on objective assessments of typical characteristics, such as 123I‐metaiodobenzylguanidine (MIBG) scintigraphy to measure cardiac autonomic dysfunction and video‐polysomnography to identify premotor rapid eye movement (REM) sleep behavior disorder (RBD).^4^ However, these assessments are costly and require specialized equipment and personnel, making it challenging to implement them for large cohort studies. Here, we used data from the Personalized Parkinson Project (PPP) cohort^5^ to evaluate whether widely accessible clinical parameters can feasibly distinguish between probable brain‐first and body‐first subtypes using a retrospective phenotyping approach.

## Methods

### Selection of Categorization Variables

We focused on assessments of typical clinical characteristics based on existing evidence and feasibility for large cohort studies. We identified three key clinical factors reflecting the proposed pathophysiological differences: (1) asymmetry of motor symptoms, reflecting the more asymmetric dopaminergic neurodegeneration expected in brain‐first PD; (2) premotor RBD, expected to be prevalent in body‐first but absent in brain‐first presentations; and (3) orthostatic hypotension (OH), a marker of autonomic dysfunction expected early in body‐first PD, but late or absent in brain‐first PD. Smell was purposely not used for classification purposes, as it may be affected early on in both phenotypes, even though the underlying pathophysiology leading to a hyposmia may be different.^6^

To assign participants to brain‐first or body‐first subtypes, we used widely available and low‐cost clinical assessments suitable for large cohort studies, with thresholds informed by existing literature and clinical expert consultation (B.B. and P.B.). Figure 1 details the proposed classification criteria. RBD was assessed using the RBD Screening Questionnaire (RBDSQ), with a score of ≤3 as a proxy for likely absence of RBD and ≥6 indicating likely presence of RBD, and scores of 4 and 5 were omitted as borderline cases. Motor asymmetry was quantified using items 3.3–3.8 and 3.15–3.17 of the Movement Disorders Society‐Unified Parkinson Disease Rating Scale (MDS‐UPDRS) part III^7^ in off, which contain separate left and right scores. The absolute difference between the summed left and right scores (|left–right|) was calculated, with a threshold of >6 (highest tertile in the PPP cohort) defining high asymmetry as expected in brain‐first PD. Because prior work^8^ has shown that most people with PD exhibit some degree of asymmetry regardless of RBD status, we propose not to be too strict regarding symmetry in the body‐first group (middle or lowest tertile). OH was determined as a binary criterion based on blood pressure measurements during the active standing test.^9^ Although OH is not very common in early PD,^10^, ^11^ it serves as an objective and specific measure (as opposed to constipation) for autonomic involvement, strengthening the validity of the body‐first subgroup. Similar to the assessment of motor asymmetry, the active standing test was performed in the off state, minimizing the short‐term effects of dopaminergic effects on OH classification. Antihypertensive medication was not stopped before the OH assessment, as this was not expected to have a major influence.^12^

**FIG. 1:**
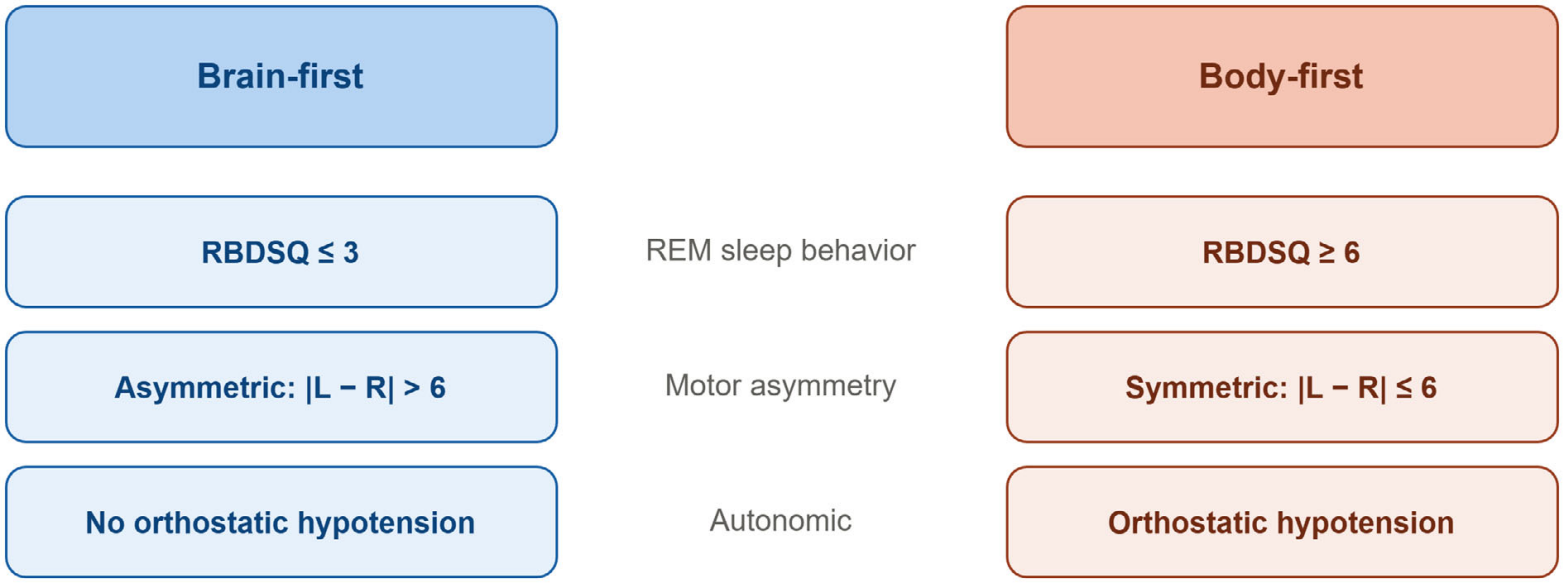
Proposed approach for distinguishing brain‐first and body‐first Parkinson's disease (PD) in large cohort studies. Motor asymmetry was calculated as the absolute difference between summed left‐ and right‐sided scores on Movement Disorders Society‐Unified Parkinson Disease Rating Scale part III items 3.3–3.8 and 3.15–3.17 (|left–right|). RBDSQ, Rapid Eye Movement (REM) Sleep Behavior Disorder Screening Questionnaire.

### Initial Validation on PPP

We applied the categorization criteria to the PPP cohort and compared the resulting probable body‐first and brain‐first subgroups on additional clinical factors expected to differ between body‐first and brain‐first PD, including constipation, cognitive impairment, and the tremor‐dominant/postural instability and gait disorder (TD/PIGD) ratio.^2^, ^13^ Constipation was assessed using items 5 to 7 of the Scales for Outcomes in Parkinson's Disease‐Autonomic (SCOPA‐AUT),^14^ and cognitive impairment was assessed using the Montreal Cognitive Assessment (MoCA).^15^ To account for differences in time since diagnosis, age, and sex, we included these variables as covariates in a multiple linear regression analysis. Because the TD/PIGD ratio shares tremor items with the asymmetry criterion, we performed a sensitivity analysis in which we removed these items from the asymmetry criterion and reclassified participants into probable body‐first and brain‐first groups. We further conducted additional exploratory analyses of gastrointestinal and urinary symptom domains, which have also been suggested as relevant to the clinical subtypes.^2^

To examine the relationship between our proposed framework and the Mild‐Motor Predominant–Intermediate–Diffuse‐Malignant (MMP‐IM‐DM) framework, we quantified the overlap in resulting classifications. Although the two frameworks are partly based on overlapping clinical variables, they differ in their conceptual aims and classification procedures.

## Results

The PPP cohort consists of 517 participants with early PD (disease duration ≤5 years).^5^ Using the criteria shown in Figure 1 and data collected during the baseline clinical visit, we selected probable brain‐first (n = 61) and body‐first (n = 35) PD participants. Table 1 shows the demographic and clinical characteristics of the whole PPP cohort and the identified subgroups. As expected, the probable brain‐first group was younger than the probable body‐first group. Furthermore, after adjusting for differences in time since diagnosis, age, and sex, probable brain‐first participants had a higher TD/PIGD ratio and showed a non‐significant tendency toward experiencing less constipation (Table 2). No differences in MoCA scores were found. After removing the tremor items from the asymmetry criterion, the difference in TD/PIGD remained (body‐first [n = 31]: 1.00 [standard deviation (SD) = 1.13]; brain‐first [n = 60]: 1.71 [SD = 1.56]; *P* = 0.01). In our exploratory analyses, the brain‐first group showed significant lower symptom burden on both total gastrointestinal and urinary symptom domains (Table S1).

**TABLE 1:** Demographic and clinical characteristics of the whole PPP cohort and the selected brain‐first and body‐first subgroups

	All (n = 517)	Brain‐first (n = 61)	Body‐first (n = 35)	*P*‐value
Demographic and general clinical characteristics at baseline				
Age (yr)	61.8 (9.1)	56.9 (8.7)	66.4 (5.7)	<0.001
Sex (number of women), n (%)	212 (41)	33 (54)	9 (26)	0.013^†^
Time since diagnosis (mo)	32.3 (17.6)	28.4 (18.2)	39.5 (19.9)	0.006
MDS‐UPDRS part III OFF score, range, 0–132	33.5 (12.9)	33.0 (10.0)	40.6 (14.5)	0.003
Clinical characteristics used for brain‐first and body‐first classification				
RBDSQ score, range, 1–13	4.7 (3.1)	2.0 (0.8)	8.6 (2.2)	
MDS‐UPDRS part III asymmetry score	4.8 (3.4)	9.3 (2.1)	2.9 (1.7)	
Orthostatic hypotension, n (%)	82 (16)	0 (0)	35 (100)	
Overlap with MMP‐IM‐DM classification^a^				
Mild‐motor predominant, n (%)	220 (43)	46 (79)	6 (18)	
Intermediate, n (%)	180 (35)	11 (19)	14 (41)	
Diffuse‐malignant, n (%)	67 (13)	1 (2)	14 (41)	

*Notes*: The *P*‐values indicate differences between the two subgroups, assessed using two‐sample *t* tests, or χ^2^ tests^†^.

Abbreviations: PPP, Personalized Parkinson Project; MDS‐UPDRS, Movement Disorder Society‐Unified Parkinson's Disease Rating Scale; RBDSQ, REM Sleep Behavior Disorder Screening Questionnaire; REM, rapid eye movement; MMP, mild‐motor predominant; IM, intermediate; DM, diffuse‐malignant.

^a^ MMP‐IM‐DM criteria were missing for 47 participants in the whole PPP cohort, for 3 in the brain‐first group and for 1 in the body‐first group.

**TABLE 2:** Initial validation of the selected brain‐first and body‐first subgroups

Dependent variable	Independent variable	β	*P*‐value	95% CI
Constipation (SCOPA‐AUT items 05–07)	**Subtype**	**−0.72**	**0.031**	**[−1.36 to −0.06]**
	Subtype	−0.69	0.083	[−1.47 to 0.09]
	Age	−0.01	0.520	[−0.05 to 0.03]
	Sex	0.39	0.221	[−0.24 to 1.03]
	**Months since diagnosis**	**0.02**	**0.004**	**[0.01–0.04]**
MOCA	Subtype	1.06	0.093	[−0.18 to 2.30]
	Subtype	0.10	0.89	[−1.39 to 1.60]
	**Age**	**−0.08**	**0.038**	**[−0.16 to −0.00]**
	Sex	1.20	0.054	[−0.02 to 2.42]
	Months since diagnosis	0.01	0.395	[−0.02 to 0.04]
TD/PIGD ratio	**Subtype**	**1.54**	**<0.001**	**[0.77–2.32]**
	**Subtype**	**1.85**	**<0.001**	**[0.90–2.79]**
	Age	0.01	0.788	[−0.04 to 0.05]
	Sex	−0.55	0.157	[−1.32 to 0.22]
	Months since diagnosis	0.01	0.437	[−0.01 to 0.03]

*Notes*: Univariable and multivariable linear regression analyses were performed using TD/PIGD ratio, MOCA and constipation as dependent variable and clinical subtype, age, sex, and disease duration as independent variables. Values in bold indicate statistically significant associations (p < 0.05).

Abbreviations: CI, confidence interval; SCOPA‐AUT, Scales for Outcomes in Parkinson's disease‐Autonomic Dysfunction; MoCA, Montreal Cognitive Assessment; TD, tremor‐dominant; PIGD, postural instability and gait difficulty.

There was some overlap between our proposed brain‐first versus body‐first classification and the MMP‐IM‐DM subtyping method (Table 1). The brain‐first group mainly consisted of mild‐motor predominant participants. However, the body‐first group overlapped not only with the diffuse‐malignant group, but also to a similar extent with the intermediate group, indicating that the body‐first and diffuse‐malignant criteria partly reflect different aspects of PD.

## Discussion

We explored a pragmatic framework to retrospectively use a brain‐first and body‐first classification in large cohort studies, in which gold‐standard measures for RBD, asymmetry in neurodegeneration, and autonomic dysfunction are not available. By applying the framework to the PPP cohort, we showed that the resulting probable brain‐first and body‐first subgroups differed as expected regarding their age and TD/PIGD ratio.^2^, ^16^, ^17^ The point estimate indicated less experienced constipation in the probable brain‐first group, although this difference was not statistically significant. However, exploratory analyses showed significantly lower total gastrointestinal and urinary symptom burden in the probable brain‐first group. It is also expected that body‐first PD participants have an increased risk of dementia compared to brain‐first participants,^2^, ^16^ but we found no differences in the MoCA score between the subgroups. This could be explained by the short disease duration of the cohort.

We found that the brain‐first group largely overlapped with the mild‐motor predominant subtype, whereas the body‐first group mainly overlapped with the non‐mild‐motor‐predominant categories, that is, with intermediate and diffuse‐malignant subtypes. This observation reinforces that early RBD and autonomic dysfunction are associated with fast progression and poor prognosis, and that body‐first etiology predisposes to fast disease progression and accelerated dementia. However, within the body‐first group, patients were distributed across both the intermediate and diffuse‐malignant subtypes. This is partly expected, as diffuse‐malignant presentations are rare in early PD cohorts.^18^ Importantly, this also reflects a conceptual difference between the two frameworks. Whereas our classification aims to capture clinical phenotypes compatible with brain‐first or body‐first origin of pathology, the MMP‐IM‐DM subtyping captures clinical phenotypes associated with the extent to which the pathology has spread. We acknowledge that clearer differences may well exist during earlier (prodromal) disease phases, when the disease pathology has not yet disseminated widely. Taken together, the present findings suggest that our proposed criteria provide another subtyping method complimentary to the MMP‐IM‐DM criteria.

Our study has several limitations. Our cohort did not have polysomnography and cardiac MIBG scintigraphy, so we lacked the gold standard for brain‐first and body‐first categorization. We also did not possess information on whether RBD symptoms had emerged during the prodromal phase, which is a prerequisite for using RBD as a marker of body‐first disease. Therefore, our findings should be interpreted with caution and the categorization criteria used here should be viewed as a tentative suggestion for further studies. Ultimately, validation of the proposed brain‐ and body‐first classification will require comparison with independent, pathological biomarkers that can provide information on the anatomical distribution of pathology. Another limitation is that a large proportion of the participants were not classified as either subtype by our proposed approach. However, this was intentional, since our goal was to identify the more extreme ends of the spectrum of brain‐first and body‐first classification, allocating participants to each of these categories with greater certainty. Both subtypes are expected to eventually converge as the disease progresses, making it increasingly difficult to distinguish them.^19^ We considered including more participants, but the advantage of greater numbers would have been offset by a diluted contrast between both groups. Therefore, the proposed criteria should only be used on recently diagnosed PD patients (here defined as ≤5 years). Nevertheless, identifying patients with a clear clinical phenotype associated with either brain‐ or body‐first PD could still provide valuable insights into differences in their longitudinal progression, and may support sample enrichment or stratification in disease‐modifying clinical trials. Predicting disease progression is an important purpose of subtyping methods.^20^ Future research could apply our pragmatic approach to cohorts with a long follow‐up duration (eg, 7–10 years^21^, ^22^) to assess its value for understanding and predicting long‐term disease progression beyond existing classification frameworks. The PPP cohort tested here will eventually also lend itself well for this purpose, as a 7‐year follow‐up assessment is ongoing. Identifying patients with a probable brain‐first or body‐first subtype could ultimately support enrichment or stratification in disease‐modifying clinical trials and, for patients who wish to receive prognostic information, contribute to counseling about their expected pattern of disease progression.

In summary, we explored a set of accessible and widely available clinical characteristics for categorizing probable brain‐ and body‐first subtypes of PD with applicability to large clinical cohorts. The resultant brain‐ and body‐first patient groups showed characteristics in line with the model, including younger age and more tremor‐dominant cases in the brain‐first group. The proposed classification partially overlapped with mild‐motor predominant and diffuse‐malignant subtypes, suggesting that the two frameworks provide complementary information. More studies are needed to refine and validate these criteria in independent cohorts and to examine their value for understanding longitudinal disease progression.

## Author Roles

Research project (1): A. Conception, B. Organization, C. Execution; Statistical Analysis (2): A. Design, B. Execution, C. Review and Critique; Manuscript Preparation (3): A. Writing of the First Draft, B. Review and Critique.

K.I.V.: 1A, 1B, 1C, 2A, 2B, 3A

N.A.T.: 1A, 1B, 1C, 2A, 2B, 3A

E.M.P.: 1A, 2C, 3B

R.C.H.: 1A, 2C, 3B

B.R.B.: 1A, 1B, 2A, 2C, 3B

P.B.: 1A, 1B, 2A, 2C, 3B

L.J.W.E.: 1A, 1B, 1C, 2A, 2C, 3B

## Disclosures

**Ethical Compliance Statement:** We confirm that we have read the Journal's position on issues involved in ethical publication and affirm that this work is consistent with those guidelines. The Personalized Parkinson Project was conducted in accordance with the Declaration of Helsinki and Good Clinical Practice guidelines, and were approved by the local medical ethics committee (Commissie Mensgebonden Onderzoek, regio Arnhem‐Nijmegen, reference number 2016‐2934). All participants provided written informed consent before enrollment at the Radboudumc.

**Financial Disclosures and Conflicts of Interest:** Author disclosures are available in the Supporting Information.

## Supporting information


**Table S1.** Exploratory analysis of the selected brain‐first and body‐first subgroups. Univariable and multivariable linear regression analyses were performed with SCOPA‐AUT gastrointestinal and urinary domain scores as dependent variable, and clinical subtype, age, sex and disease duration as independent variables. SCOPA‐AUT, scales for outcomes in Parkinson's disease‐autonomic dysfunction.

**Data S1.** Disclosure Statement form.

## Data Availability

Data from the Personalized Parkinson Project used in the present study were retrieved from the PEP database (https://pep.cs.ru.nl/index.html). The PPP data is available on request via: ppp-data@radboudumc.nl. More details on the procedure can be found on the website www.personalizedparkinsonproject.com/home.

## References

[1] Borghammer P , Van Den Berge N . Brain‐first versus gut‐first Parkinson's disease: a hypothesis. J Parkinsons Dis 2019;9:S281–S295. 10.3233/JPD-191721. PMC6839496 31498132

[2] Horsager J , Borghammer P . Brain‐first vs. body‐first Parkinson's disease: an update on recent evidence. Parkinsonism Relat Disord 2024;122:106101. 10.1016/j.parkreldis.2024.106101 38519273

[3] Xu Z , Hu T , Xu C , et al. Disease progression in proposed brain‐first and body‐first Parkinson's disease subtypes. NPJ Park Dis 2024;10:111. 10.1038/s41531-024-00730-1 PMC11150376 38834646

[4] Pitton Rissardo J , Fornari Caprara AL . Cardiac 123I‐Metaiodobenzylguanidine (MIBG) scintigraphy in Parkinson's disease: a comprehensive review. Brain Sci 2023;13:13. 10.3390/BRAINSCI13101471. PMC10605004 37891838

[5] Bloem BR , Marks WJ , Silva de Lima AL , et al. The personalized Parkinson project: examining disease progression through broad biomarkers in early Parkinson's disease. BMC Neurol 2019;19:160. 10.1186/s12883-019-1394-3 PMC6636112 31315608

[6] Lee W‐W . Revisiting PD: unraveling the brain‐first and body‐first perspectives. Ann Clin Neurophysiol 2024;26:39–45.

[7] Goetz CG , Tilley BC , Shaftman SR , et al. Movement Disorder Society‐sponsored revision of the unified Parkinson's disease rating scale (MDS‐UPDRS): scale presentation and clinimetric testing results. Mov Disord 2008;23:2129–2170. 10.1002/mds.22340 19025984

[8] Lövdal SS , Carli G , Orso B , et al. Investigating the aspect of asymmetry in brain‐first versus body‐first Parkinson's disease. NPJ Park Dis 2024;10:74. 10.1038/s41531-024-00685-3 PMC10981719 38555343

[9] Brignole M , Moya A , De Lange FJ , et al. ESC guidelines for the diagnosis and management of syncope. Eur Heart J 2018;39:1883–1948. 10.1093/eurheartj/ehy037 29562304

[10] Velseboer DC , de Haan RJ , Wieling W , Goldstein DS , de Bie RMA . Prevalence of orthostatic hypotension in Parkinson's disease: a systematic review and meta‐analysis. Parkinsonism Relat Disord 2011;17:724–729. 10.1016/j.parkreldis.2011.04.016 PMC5199613 21571570

[11] Baschieri F , Sambati L , Guaraldi P , Barletta G , Cortelli P , Calandra‐Buonaura G . Neurogenic orthostatic hypotension in early stage Parkinson's disease: new insights from the first 105 patients of the BoProPark study. Parkinsonism Relat Disord 2021;93:12–18. 10.1016/j.parkreldis.2021.11.002 34758441

[12] Bhanu C , Nimmons D , Petersen I , et al. Drug‐induced orthostatic hypotension: a systematic review and meta‐analysis of randomised controlled trials. PLoS Med 2021;18:e1003821. 10.1371/journal.pmed.1003821 PMC8577726 34752479

[13] Stebbins GT , Goetz CG , Burn DJ , Jankovic J , Khoo TK , Tilley BC . How to identify tremor dominant and postural instability/gait difficulty groups with the movement disorder society unified Parkinson's disease rating scale: comparison with the unified Parkinson's disease rating scale. Mov Disord 2013;28:668–670. 10.1002/mds.25383 23408503

[14] Visser M , Marinus J , Stiggelbout AM , van Hilten JJ . Assessment of autonomic dysfunction in Parkinson's disease: the SCOPA‐AUT. Mov Disord 2004;19:1306–1312. 10.1002/mds.20153 15390007

[15] Nasreddine ZS , Phillips NA , Bédirian V , et al. The Montreal cognitive assessment, MoCA: a brief screening tool for mild cognitive impairment. J Am Geriatr Soc 2005;53:695–699. 10.1111/j.1532-5415.2005.53221.x 15817019

[16] Borghammer P . The brain‐first vs. body‐first model of Parkinson's disease with comparison to alternative models. J Neural Transm 2023;130:737–753. 10.1007/s00702-023-02633-6 37062013

[17] Boertien J , Nazmuddin M , Kłos J , et al. Striatal dopaminergic asymmetry as a marker of brain‐first and body‐first subtypes in de novo Parkinson's Diseas. medRxiv 2022. 10.1101/2022.12.23.22283910.

[18] Fereshtehnejad SM , Zeighami Y , Dagher A , Postuma RB . Clinical criteria for subtyping Parkinson's disease: biomarkers and longitudinal progression. Brain 2017;140:1959–1976. 10.1093/brain/awx118 28549077

[19] Borghammer P , Horsager J , Andersen K , et al. Neuropathological evidence of body‐first vs. brain‐first Lewy body disease. Neurobiol Dis 2021;161:105557. 10.1016/j.nbd.2021.105557 34763110

[20] Marras C , Fereshtehnejad S‐M , Berg D , et al. Transitioning from subtyping to precision medicine in Parkinson's disease: a purpose‐driven approach. Mov Disord 2024;39:462–471. 10.1002/mds.29708 38243775

[21] Fereshtehnejad S‐M , Moqadam R , Azizi H , et al. Distinct longitudinal clinical‐neuroanatomical trajectories in Parkinson's disease clinical subtypes: insight toward precision medicine. Mov Disord 2025;40:1572–1583. 10.1002/mds.30229 PMC12371632 40415649

[22] Negida A , Mukhopadhyay N , Berman BD , Barrett MJ . Comparative analysis of progression milestones across Parkinson's disease clinical, pathological, and data‐driven subtypes: a 10‐year follow‐up. Npj Park Dis 2026;12:239. 10.1038/s41531-026-01430-8 PMC13616926 42286020

