# Supplementary material for: A Pragmatic Approach for Brain‐First and Body‐First Classification in Parkinson's Disease: Insights from the Personalized Parkinson Project
Source: Mov Disord Clin Pract. 2026 Nov 9:10.1002/mdc3.70913. Online ahead of print. doi: 10.1002/mdc3.70913 (PMC13746290; doi:10.1002/mdc3.70913)
Supplement: Supplementary file 2 [file MDC3-9999-0-s002.docx]

*Supplementary Table 1: Exploratory analysis of the selected brain-first and body-first subgroups. Univariable and multivariable linear regression analyses were performed with SCOPA-AUT gastrointestinal and urinary domain scores as dependent variable, and clinical subtype, age, sex and disease duration as independent variables. SCOPA-AUT: Scales for Outcomes in Parkinson's disease - Autonomic Dysfunction.*

| Dependent variable | Indepedent  variable | Beta | P-value | 95% CI |
| --- | --- | --- | --- | --- |
| Gastrointestinal symptoms  (SCOPA-AUT items 01-07) | Subtype | -1.84 | <0.001 | [-2.82, -0.86] |
|  | Subtype | -1.91 | 0.001 | [-3.08, -0.73] |
|  | Age | -0.03 | 0.261 | [-0.09, 0.03] |
|  | Sex | 0.50 | 0.298 | [-0.45, 1.46] |
|  | Months since diagnosis | 0.04 | 0.004 | [0.01, 0.06] |
| Urinary symptoms  (SCOPA-AUT items 08-13) | Subtype | -3.98 | <0.001 | [-5.47, -2.50] |
|  | Subtype | -3.01 | 0.001 | [-4.80, -1.23] |
|  | Age | 0.09 | 0.054 | [-0.00, 0.18] |
|  | Sex | 1.01 | 0.170 | [-0.44, 2.46] |
|  | Months since diagnosis | 0.04 | 0.049 | [0.00, 0.07] |
